# Computational Biology Dynamics of Mps1 Kinase Molecular Interactions with Isoflavones Reveals a Chemical Scaffold with Potential to Develop New Therapeutics for the Treatment of Cancer

**DOI:** 10.3390/ijms232214228

**Published:** 2022-11-17

**Authors:** Lauren Pugh, Alisha Pancholi, Priscila Celeste Purat, Sandra Agudo-Alvarez, Raúl Benito-Arenas, Agatha Bastida, Victor M. Bolanos-Garcia

**Affiliations:** 1 Department of Biological and Medical Sciences, Faculty of Health and Life Sciences, Oxford Brookes University, Gipsy Lane, Headington, Oxford OX3 0BP, UK; 2 Departamento de Química Bio-Orgánica, IQOG, c/Juan de la Cierva 3, E-28006 Madrid, Spain

**Keywords:** Mps1, cancer, poor prognosis tumours, flavonoid-like compounds, bioinformatics, molecular docking

## Abstract

The protein kinase Mps1 (monopolar spindle 1) is an important regulator of the Spindle Assembly Checkpoint (SAC), the evolutionary conserved checkpoint system of higher organisms that monitors the proper bipolar attachment of all chromosomes to the mitotic spindle during cell division. Defects in the catalytic activity and the transcription regulation of Mps1 are associated with genome instability, aneuploidy, and cancer. Moreover, multiple Mps1 missense and frameshift mutations have been reported in a wide range of types of cancer of different tissue origin. Due to these features, Mps1 arises as one promising drug target for cancer therapy. In this contribution, we developed a computational biology approach to study the dynamics of human Mps1 kinase interaction with isoflavones, a class of natural flavonoids, and compared their predicted mode of binding with that observed in the crystal structure of Mps1 in complex with reversine, a small-sized inhibitor of Mps1 and Aurora B kinases. We concluded that isoflavones define a chemical scaffold that can be used to develop new Mps1 inhibitors for the treatment of cancer associated with Mps1 amplification and aberrant chromosome segregation. In a broader context, the present report illustrates how modern chemoinformatics approaches can accelerate drug development in oncology.

## 1. Introduction

Cancer has become the second leading cause of death in the human population, contributing to one in six deaths worldwide. According to the World Health Organisation (WHO), the most common cancers are breast, lung, prostate, stomach, and colorectal [1]. Current treatments for cancer can be quite ineffective for a number of reasons, ranging from the severe side effects of current chemotherapies to the rapid acquisition of drug resistance by the treated cancer cells [2]. Diverse protein kinases such as Aurora B, CDK1, CK2, PLK1, and Mps1 contribute importantly to the control of cell division [3,4,5,6,7]. The latter enzyme, monopolar spindle 1 kinase (Mps1), also known as TTK, is a versatile kinase with essential roles in the regulation of chromosome segregation, centromeres duplication and accurate chromosome alignment during mitosis [8,9,10]. Mps1 is a central protein component and upstream regulator of the Spindle Assembly Checkpoint (SAC), the evolutionary conserved self-surveillance signalling system of higher organisms that monitors the proper segregation of chromosomes during mitosis and meiosis. Mps1 inhibition prevents recruitment of other SAC proteins to the kinetochore, impairing the transition of metaphase to anaphase [11]. Mps1 is overexpressed in a range of tumours of different tissue origin including bladder, breast, esophagus, lung, prostate and thyroid and it is a driver of tumour progression [12,13]. Given the prominence of Mps1 association with oncogenesis, this kinase has arisen as a bona fide target for the treatment of cancer.

**Figure 1:**
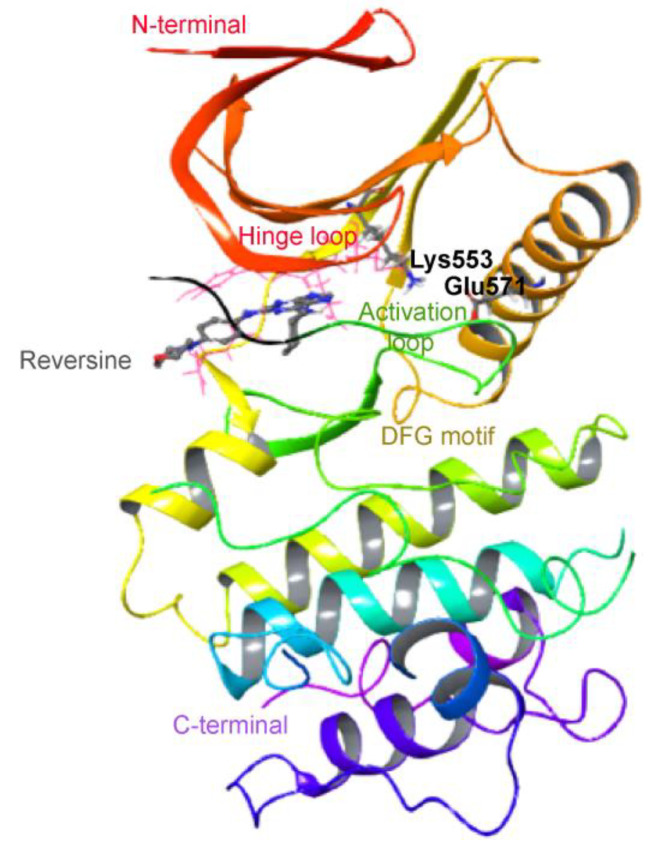
Structure of Mps1 kinase domain (PDB ID 5ljj).

Mps1 is a protein organised into two main domains, an N-terminal region harbouring a triple tandem arrangement of the tetratricopeptide repeat (TPR) motif and a C-terminal region that harbours the characteristic bi-lobal fold of a protein kinase [14,15,16]. In human Mps1, the latter domain is defined by an N-terminal lobe composed of several α-helices and six β-strands, spanning residues Glu516 to Met602 (Figure 1) while its C-terminal lobe accommodates numerous α-helices, ranging from Asn606 to Gln794. The two lobes of the kinase domain are connected by a short hinge loop involving residues Glu603 to Gly605.

In human Mps1, the activation loop is defined by residues Gln672 to Lys680 and this region is only partially visible in refined crystal structures due to its intrinsic flexibility [16]. The adoption of an extended conformation allows access of the substrate ATP to the cleft of the active site, creating a substrate beheading interface at the C terminal area of the activation loop. Phosphorylation of this segment is required for Mps1 full enzyme activity. The orientation of the Lys553 residue is important for the alignment of the adenosine triphosphate and the transfer and catalysis of the γ-phosphate to the substrate. To maintain the correct lysine orientation, the catalytic alpha helix establishes an ionic interaction with residue Glu571. The activation loop contains a conserved DFG (Asp-Phe-Gly) motif, which is crucial to the regulation of Mps1 kinase activity and displays two different conformations, “In” and “Out.” Flipping the DFG motive moves the aspartate residue away from the ATP binding side, eventually leading to an inactive state termed “DFG out”. “DFG out” unlocks a new allosteric pocket adjacent to the ATP binding pocket. Due to these features, in principle, manipulation of such a dynamic conformational transition represents an opportunity for the development of more selective Mps1 kinase inhibitors [17].

Multiple small-sized inhibitors of Mps1 catalytic activity have been reported in recent years [18,19,20,21,22,23]. From a drug development perspective, determining exactly how the various inhibitors bind to the Mps1 catalytic domain at the atom resolution level is important in order to to design better drugs to treat cancers of poor prognosis such as liver caner, which has shown great resilience to multiple types of drugs. The current chemotherapies used to treat liver cancer include but are not limited to carboplatin and doxorubicin. Unfortunately, these drugs are only effective against a specific portion of tumours, with an overall response in patients lasting for a short period of time [24]. However, an independent study reported the use of the Mps1 inhibitor TC mps1 12 on human hepatocellular carcinoma (HCC), which resulted in gross centrosome disorganisation and chromosome misalignment, leading to a shortened mitotic duration and, ultimately, to apoptosis [9]. These findings add support to the notion that Mps1 inhibition offers great potential to develop more effective chemotherapies for the treatment of liver cancer and possibly other forms of aggressive cancer of high mortality and different tissue origin.

Although currently no drugs to treat cancer that target Mps1 catalytic activity have been approved for their use in the clinic, some Mps1 kinase inhibitors have been advanced to clinical trials, such as the compounds termed BAY 1217389 and BAY 1161909 [25,26]. These compounds were developed by Bayer AG and the clinical trial included the use of these compounds in combination with paclitaxel, an approved drug to treat lung and breast cancer [26]. Phase I studies of orally administered BAY 1217389 in combination with intravenous paclitaxel were completed last year (clinical trials USA government identifier NCT02366949 and NCT02138812, respectively). The results of these trials seemed encouraging, as these compounds were reported to exhibit good efficacy and relatively moderate adverse effects such as fatigue and anaemia. However, due to a strategic decision, only the latter compound was recommended to be advanced to Phase II studies in combination with paclitaxel in patients with advanced malignancies. The other small-sized inhibitors of Mps1 kinase that have reached clinical trials are BOS172722 [27] and CFI-402257 [28]. BOS172722 was used in combination with paclitaxel in a Phase I study conducted in patients with advanced non-haematologic malignancies. An example of such a Phase I study was completed early last year (clinical trials USA government identifier NCT03328494), but no details of this study have been disclosed to date. The compound CFI-402257 has also been subjected to clinical trials. This includes an ongoing Phase I/Phase II study where this molecule is used in combination with paclitaxel in patients with advanced HER2-negative breast cancer (clinical trials USA government identifier NCT03568422) and an independent study where CFI-402257 has been used in combination with the drug fulvestrant in breast cancer patients (clinical trials USA government identifier NCT05251714). A third, independent, Phase I study is currently investigating the use of CFI-402257 in patients with advanced solid tumours (clinical trials USA government identifier NCT02792465).

Unfortunately, the traditional route for drug discovery and development remains expensive and time consuming, with an average timeframe of 10 to 15 years of intensive work required before the drug reaches the market [29]. Yet, the success rate of drugs that reached clinical trials during 2006 to 2015 was only 9.6% [30]. In recent years, computational biology has been raised as a powerful tool that enables a more rapid design and identification of novel chemical entities of potential therapeutic value against a wide range of chronic and infectious diseases [31,32,33,34,35]. Computer-aided drug discovery spans ligand-based and target structure-based drug design as well as the in silico screening of ADMET (adsorption, distribution, metabolism, excretion and toxicity) pharmacological properties at the early stage of drug development. In principle, these advances can lead to a more rapid identification and validation of novel potential drug candidates [36]. The promise that computer-aided drug discovery represents is reflected in the exponential growth in the use of artificial intelligence and machine learning approaches currently adopted by new and well-established biotech and pharma companies around the globe. Of course, the compounds designed using a computational biology approach must be tested experimentally to assess their true therapeutic activity. Well-established biological tests that are routinely used for this include cytotoxicity and clonogenic assays against a panel of cancer cell lines. One widely used test to determine the cytotoxicity of drugs and drug-like compounds is the MTT assay, a colorimetric assay that determines the relative number of live cells from their metabolic activity. The assay is based on the conversion of the water-soluble substrate MTT (3-(4,5-dimethylthiazol-2-yl)-2,5-diphenyltetrazolium bromide) to formazan, an insoluble product of purple colour, by the action of mitochondrial reductase [37]. Only viable cells with active metabolism convert MTT into formazan. Hence, colour formation serves as a marker of cell viability and toxicity upon the treatment of the cells with new and established drugs and/or other biological active molecules such as therapeutic antibodies.

The clonogenic assay, also known as colony formation assay, is an in vitro cell survival assay that is based on the ability of a single cell to grow into a colony [38]. This type of assay is commonly used to determine the effect of ionising radiation and cytotoxic molecules, in which case only a fraction of seeded cells will retain the capacity to produce colonies. Colonies are fixed with glutaraldehyde and stained with crystal violet before being counted under a microscope [38].

One aspect of cancer research that deserves more attention is the significant differences in cancer incidence in different ethnic populations. In addition to underlying genetic factors, there is mounting evidence suggesting that diet can play an important role in oncogenesis. For example, Asian populations consume a significantly higher soy food diet compared to Western populations [39] and this seems to correlate with the comparatively much lower incidence of cancer in the former population [40]. A soy diet is of particular interest because soy products are abundant sources of the isoflavones genistein (4′,5,7-trihydroxyisoflavone), daidzein (7,4′-dihydroxyisoflavone) and glycitein (7,4′-dihydroxy-6-methoxyisoflavone) [41,42,43]. These types of flavonoids are a potential chemical class of compounds from which anti-cancer drugs can be developed [44,45]. This notion is supported by previous independent studies where certain isoflavones were tested in breast cancer survivors as a form of prevention treatment for the recurrence of cancer [46]; the discovery that the flavanol fisetin interacts with cyclin-dependent kinase 6 [47]; and the observation that genistein, the most abundant isoflavone found in soy products, is an inhibitor of the protein tyrosine kinase (PTK), halting the proliferation of cancerous cells [48,49]. Genistein has also been shown to interact with death-associated protein kinase 1 (DAPK1) and has been suggested as a potential treatment for endometrial adenocarcinomas [50]. Because genistein may be able to interact and inhibit other protein kinases that are upregulated and/or overexpressed in cancer, this molecule represents an interesting chemical scaffold from which more specific, novel cancer therapeutics can be developed.

Moreover, it is encouraging that daidzein and glycitein, when administered alongside tamoxifen (an anti-oestrogen medication that blocks the activity of oestrogen and is prescribed to breast cancer survivors) may decrease the risk of cancer recurrence [51]. These findings prompted us to develop a computational biology approach to investigate the potential molecular interactions between human Mps1 kinase and certain isoflavones and to compare the dynamics of such interactions against those of Mps1 with reversine. In this way, we identified certain isoflavones that are predicted to physically interact with the human Mps1 kinase domain with good binding affinity, strongly suggesting that these molecules correspond to a chemical class of small-sized compounds that can be used as lead chemotypes to design and develop more effective Mps1 inhibitors for the treatment of cancer.

## 2. Results

### 2.1. Virtual Screening of Mps1 Using Reversine as Lead Compound

Mps1 inhibitors described in the literature are ATP competitive inhibitors. Most of them present adenine moiety and establish between 1–3 hydrogen bonds with amino acid residues that define the hinge region (e.g., residues Met602-Asn606). In the first place, molecular modeling studies of the Mps1 kinase domain with some of the most effective inhibitors described in the literature (Table 1) were carried out to determine which one to use as the lead compound for HTVS analysis.

**Figure 2:**
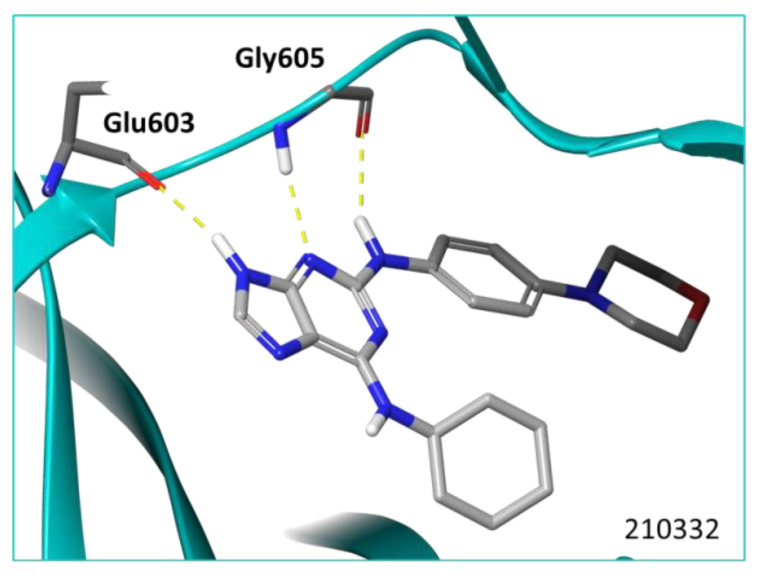
Reversine can establish polar interactions with the Mps1 kinase domain.

Reversine is a small molecule with high affinity for the ATP site binding of Mps1. This interaction is stabilised through the establishment of three hydrogen bond interactions with the hinge loop residues (two H-bonds with Gly605 and one with Glu603) (Figure 2 and Supplementary Figure S1a).

However, reversine is a small molecule first reported as an inhibitor of Aurora B kinase that abolished the Aurora B-dependent phosphorylation of histone H3. Hence, the search for more selective inhibitors of Mps1 remains important. To this aim, we first carried out the exploration of a chemical library of compounds that are structurally related to reversine (CID 210332). For this, the PubChem database with a 0.9 Tanimoto similarity index was screened. Upon conformational analyses, all the compounds were subjected to the high-throughput virtual screening (HTVS) XP Glide docking protocol. Compounds with docking Glide scores better than −9.0 kcal/mol, predicted specific interactions with Gly605-Glu603 residues, and significant structural diversity were considered the most promising Mps1 kinase ligands (Table 2).

**Figure 3:**
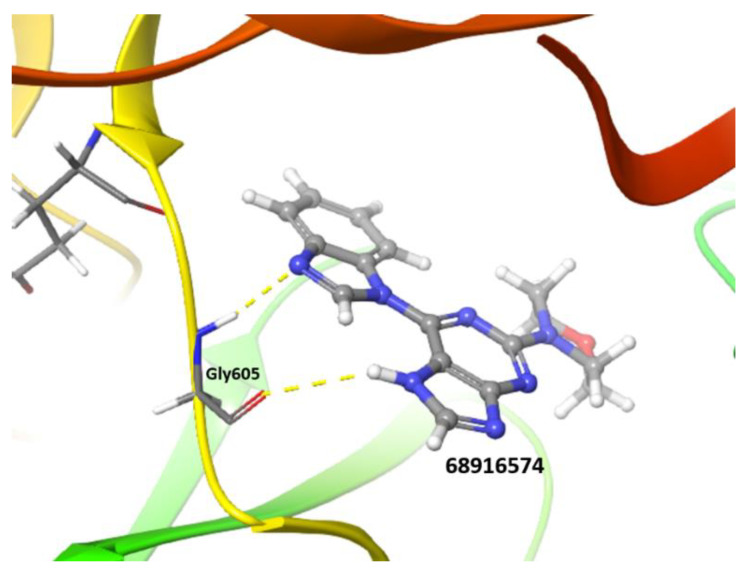
Cartoon showing the structure of the Mps1 kinase–68916574 small compound complex. The latter molecule is shown in grey (XP GScore −9.3 kcal/mol). H-bond interactions between 68916574 and the Mps1 residue Gly605 are shown in yellow dashed lines.

Additional criteria to further filter out candidates included the computational analysis of predicted ADME properties and the identification of potential structural promiscuous moieties or PAINS (pan-assay interference compounds) [55,56] for each molecule. To this aim, a set of 34 chemical descriptors were calculated using the QikProp module for the best compounds obtained by XP GScore. The swissADME webserver was used to identify structural alerts for each chemotype (Table 3). These analyses showed that compound 68916574 exhibits the best physicochemical properties and no PAINS features. Importantly, in addition to a high docking energy score, the compound 68916574 is predicted to have a good druggability profile when compared to reversine. Taken together, these analyses strongly suggest that compound 68916574 harbours the favourable structural, physicochemical, and pharmacological properties required for drug development (Figure 3).

### 2.2. Virtual Screening of Mps1 Using Genistein as the Lead Compound

**Figure 4:**
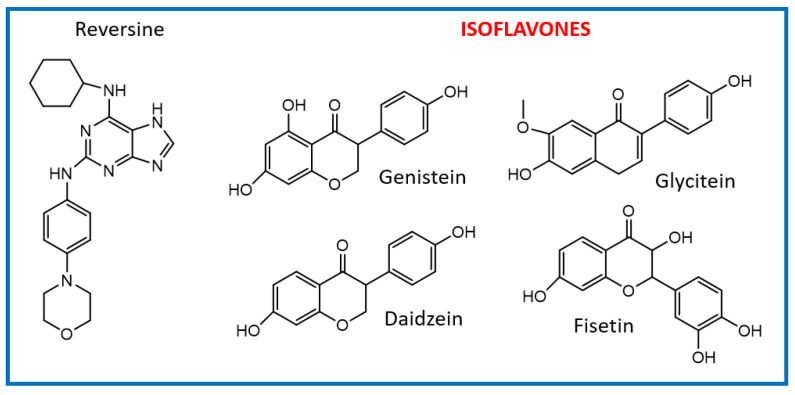
Comparison of the chemical structures of the isoflavones daidzein, fisetin, genistein, and glycitein and the Mps1 inhibitor reversine.

The isoflavone genistein has been reported to interact with death-associated protein kinase 1 (DAPK1), and the structure of genistein in complex with death-associated protein kinase 1 (PDB ID 5auz) has been solved at high resolution (e.g., 1.60 Å) [50]. Another flavonoid, fisetin, has been reported to induce mitotic arrest in a proteasome-dependent manner [57] through its binding and subsequent inhibition of protein kinase cyclin-dependent kinase 6. Atomic details of this interaction are known as the structure of fisetin in complex with human cyclin-dependent kinase 6 (PDB ID 1xo2) has been reported [47]. Fisetin, genistein, daidzein, and glycitein share important chemical structure similarities, adding support to the notion that genistein-based compounds, including other isoflavones, may be used as molecular scaffolds to develop novel Mps1 kinase inhibitors of therapeutic importance in oncology (Figure 4).

**Figure 5:**
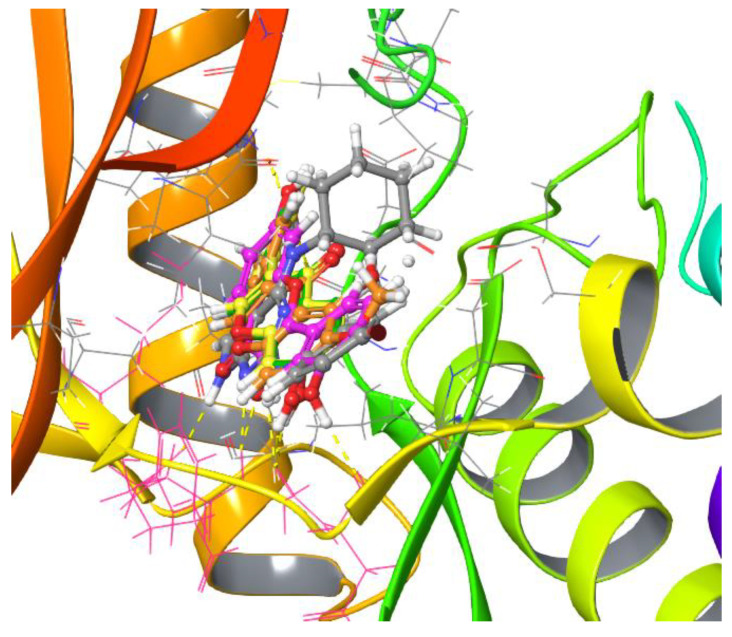
Details of the interaction between the human Mps1 kinase domain and reversine (grey) (PDB ID 5ljj) and its comparison with the anticipated mode of binding to Mps1 of the flavonoids fisetin (pink), genistein (green), daidzein (yellow), and glycitein (orange) as predicted by molecular docking. H-bond interactions are shown in yellow dashed lines. Details of these interactions are also shown in Supplementary Figure S1b–d, respectively.

The mode of interaction of the Mps1 kinase domain bound to reversine (PDB ID 5ljj) was compared against the predicted mode of interaction with the isoflavonoids daidzein, genistein, glycitein, and fisetin (Figure 5).

The predicted best energy of binding was for the following flavonoids: fisetin (−10.27 kcal/mol) and genistein (−9.7 kcal/mol). For comparison, the predicted best energy of binding for reversine was −10. 9 kcal/mol. Fisetin is predicted to establish 2 H-bonds with the amino acid residues Asn606 and Gly605 whereas the other three flavonoids are predicted to establish only one H-bond with residue Gly605. Because fisetin exhibited violation of the Lipinski rules, it was excluded from the selection process. Genistein was chosen as the best isoflavonoid as it presents a good Glide score as well as predicted physicochemical and pharmacological properties (see Table 4 for details).

**Figure 6:**
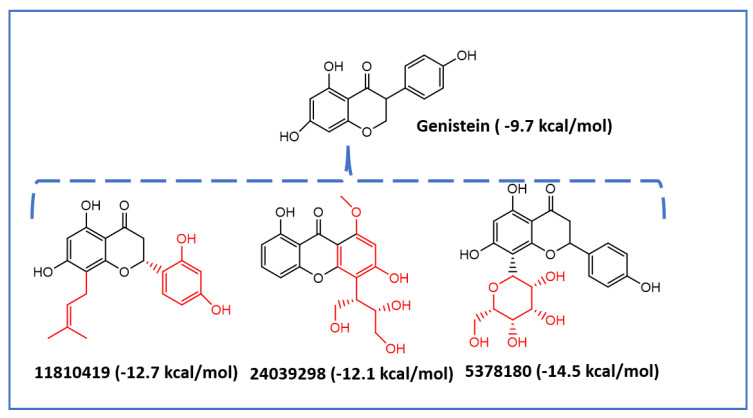
Structural diversity of new isoflavoids as potential Mps1 kinase inhibitors. The number in brackets corresponds to the Glide score.

**Figure 7:**
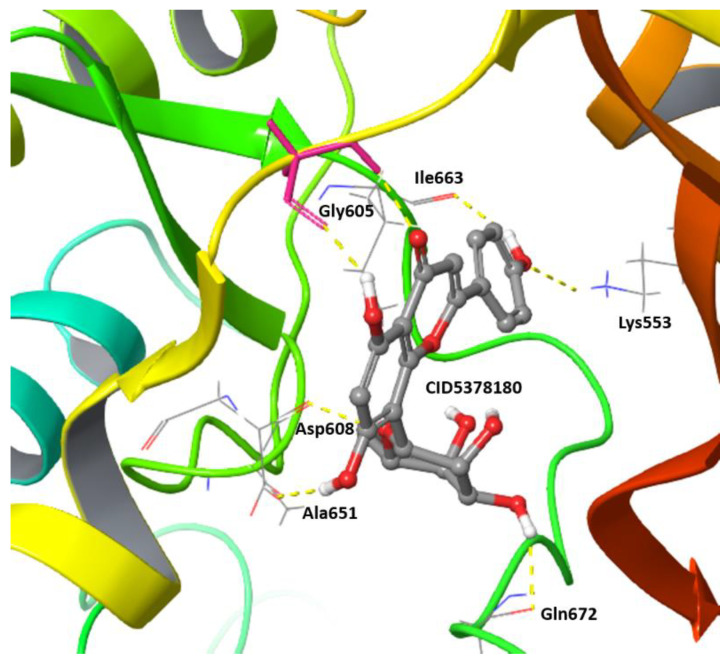
Molecular docking of the top selected isoflavonid, the compound 5378180, to human Mps1 kinase domain (PDB ID 5ljj). The protein is shown in cartoon representation and the compound 5378180 in stick-ball representation. H-bond interactions are shown in yellow dashed lines (Gly605, Asp608, Ala651, Gln672, Ileu663 and Lys553). The Glide score of this interaction was −14.5 kcal/mol.

Genistein (CID 5280961) was used to identify new potential flavonoids as possible Mps1 kinase inhibitors from a chemical library of compounds (e.g., the PubChem database) that are structurally related to this molecule. More than one thousand compounds were screened and analysed. Upon conformational analyses, all the compounds were subjected to the high-throughput virtual screening (HTVS) Glide docking protocol and then XP docking Glide scores better than −9.0 kcal/mol, and those that presented specific interactions with Gly605-Glu603 residues and showed chemical structure diversity were considered for selecting the most promising Mps1 kinase binders (117 compounds in total). The structural diversity of the best compounds obtained by molecular docking as potential Mps1 kinase inhibitors is presented in Figure 6. Molecular docking results showed that all of the compounds fit well in the adenine site of the Mps1 kinase domain. The molecular interaction of the best isoflavonoid (CID5378180) showed seven hydrogen bonds with the Gly605, Asp608, Ala651, Gln672, Lys553, and Ile663 residues and a predicted binding energy of −14.5 kcal/mol (genistein −9.7 kcal/mol) (Figure 7).

To further filter out candidates, ADMET (absorption, distribution, metabolism, excretion and toxicity) and PAINS (pan-assay interference compounds) were investigated for each flavonoid (see Table 5 for details). 

Based on the aforementioned selection criteria, the compounds CID5378180, CID11810419, and CID24039298 were identified as the most promising flavonoid-based compounds to develop new drug candidates to target Mps1 catalytic activity.

## 3. Discussion

This research aimed to explore the molecular details of the interaction between certain flavonoid molecules, specifically between the subclass isoflavones and the human protein kinase Mps1 using a computational biology approach. We also critically analysed the potential use of this chemical class of compounds as a source of molecular scaffolds to develop new anti-cancer drugs taking into consideration pharmacological properties predicted in silico.

Mps1 overexpression has been reported in aggressive types of cancer of poor prognosis such as glioblastomas, triple-negative breast cancer and pancreatic cancer [59,60] and it has been suggested that this may be due to abnormal activation of the SAC and the induction of genome instability [59]. Hence, inhibition of Mps1 catalytic activity can lead to better therapeutics for cancers that do not respond well to current treatment options. Structural details of the ATP binding pocket and the Mps1 kinase in complex with ATP are shown in Supplementary Figure S2a,b, respectively. Indeed, several small-sized compounds that act as ATP binding competitors in a range of tumour cells of diverse tissue origin have been reported in the literature. One example is the over expression of Mps1 in neuroblastoma patients, which has been linked to poor prognosis and tumour progression [60]. Importantly, inhibition of Mps1 catalytic activity by the small molecule inhibitor reversine in neuroblastoma cells results in the halt of cell proliferation and the induction of mitochondrial apoptosis [60,61]. However, it is important to bear in mind that reversine was originally reported as an inhibitor of Aurora B. Subsequent studies showed that reversine exhibits a two-fold higher affinity to Mps1 than Aurora B [62], which limits the use of this small-sized compound in the clinic. Such important disadvantage has stimulated the search of more specific inhibitors of Mps1 kinase activity, such as the compound CFI-402257, which prevents activation of the SAC, impacting cancer cells survival by preventing cells progression to anaphase and inducing cell death in breast, colorectal, pancreatic, lung, and prostate cancer cell lines (Table 1) [63]. However, success of CFI-402257 in the clinic is highly questionable due to the observation of severe side effects such as damage to the gastrointestinal tract and haematological changes [63].

More recently, RMS-07, a synthetic compound derived from a core scaffold of the Mps1 binding site, was reported as a potential new chemotherapy to treat triple-negative breast cancer [64]. One interesting feature of compound RMS-07 is its underlying mechanism of action, involving an irreversible covalent interaction with Mps1. This mode of binding is achieved through the targeting of a cysteine residue of low conservation that is located in the hinge region of the Mps1 kinase domain [64]. It would be very interesting to know how well this compound performs in the successive clinical trials. Taken together, these findings clearly indicate the need to develop better Mps1 kinase inhibitors to more effectively tackle cancers of poor prognosis and different tissue origin. In response to this health challenge of global proportions, we explored the use of natural flavonoids for the development of anti-cancer therapeutics, an area of increasing interest in cancer therapy. This approach offers unique advantages such as the molecules being readily available and their isolation being inexpensive when compared to the generation of synthetic drugs. Moreover, natural molecules have an evolutionarily optimised structure which often confers additional benefits such as higher molecular rigidity when compared to synthetic compounds [65]. Furthermore, natural flavonoid compounds have been widely researched for their involvement in signal transduction within cancer cells, with recent findings suggesting that they have anti-cancer abilities through promoting apoptosis and inhibiting cell proliferation and angiogenesis [66]. Indeed, certain types of natural flavonoids and derived semi-synthetic compounds have been shown to act as effective inhibitors of protein kinases. One example is flavopiridol, a semi-synthetic flavonoid that is in clinical trials and proposed for the treatment of cancer. This compound is a derivative of a natural compound produced by an Indian plant, in which the natural flavone was modified at its 3-hydrox-1-methylpiperidinyl (D-ring) to increase its inhibitor activity of cyclin-dependent kinase 9 [67,68]. A second example is the interaction of genistein with cyclin-dependent kinase 6 (CDK6), which involves the establishment of three polar interactions with CDK6.

The current study presents a computational biology approach to show that compound 68916574 raises as an interesting chemical scaffold to design and develop a new generation of human Mps1 kinase inhibitors. Structural details of the compound 68916574-Mps1 kinase interaction are shown in Supplementary Figure S3a. Interestingly, our docking analysis predicted that genistein can establish a similar number of polar interactions with Mps1. We argue that the nature and extent of the stabilising interactions between Mps1 and genistein represents a new molecular scaffold to develop new semi-synthetic compounds to tackle aggressive cancer types associated with chromosome segregation defects and/or where Mps1 kinase is amplified or constitutively active. Future work will aim to validate the chemoinformatics analyses described in this contribution using an experimental approach that includes the quantification of the binding affinity of small-sized inhibitors for Mps1 kinase using biophysical methods such as isothermal titration calorimetry (ITC), surface plasmon resonance (SPR) and/or microscale thermophoresis (MST), to name a few, as well as cytotoxic, clonogenic, and functional assays of Mps1 kinase activity inhibition to establish the relative potency of the compounds.

Another isoflavone, glycitein, has been suggested to have a role in inhibiting tumourigenesis of the breast cancer cell lines MDA-MB-23 and SKBR-3 [69]. However, glycitein’s ability to inhibit tumourigenesis is weak and requires high concentrations to receive minimal benefits. Glycitein’s mechanism of action is through DNA synthesis rather than acting as a kinase inhibitor [69]. Nevertheless, it seems worth further exploring the molecular details of the mode of action of this isoflavone as it may represent yet another opportunity to develop anti-cancer therapies beyond the inhibition of protein kinases.

With >60% of medicines being derived from natural products or secondary metabolites of such products, the generation of novel therapeutics based on exploring natural products remains important. Further investigations that include the use of functional assays will aim to establish if genistein and the small-sized compounds CID5378180, CID11810419, and CID24039298 that we identified using a chemoinformatics approach, constitute suitable chemotypes to develop more potent and specific Mps1 kinase inhibitors. Structural details of the predicted mode of binding of these compounds to Mps1 kinase domain are shown in Supplementary Figure S3b–d, respectively. Moreover, for specific types of cancer it may be possible to use new Mps1 inhibitors derived from the aforementioned chemotypes alone to replace the current chemotherapies or to be used in conjunction with other drugs and/or radiotherapy for the treatment of aggressive tumours of poor prognosis.

## 4. Materials and Methods

### 4.1. Ligand Receptor Structure

A 3D structure model of the human Mps1 kinase domain based on the crystal structure of this enzyme in complex with reversine and deposited in the Protein Data Bank (PDB) under the ID 5LJJ [52] was used to dock potential ligands, including isoflavones and flavonoid-like compounds. To this aim, protein optimisation of the chosen Mps1 3D structure was first carried out using the Protein Preparation Wizard module, which is included in the Maestro suite software (Schrödinger, LLC, New York, NY, USA, 2020).

Reversine and the isoflavonoid genistein were employed to retrieve structurally similar compounds from PubChem [70]. The criteria used for this was a Tanimoto similarity index >90%, and the following filters: molecular weight 270–500 Da; a 1–9 routable bond count; 1–7 H-bond donors; 3–11 H-bond acceptors; a 19–37 heavy atom count; and a 1–7 xLog(P). A total of 486 Mps1 compounds based on the chemical structure of reversine and 1000 compounds based on genistein chemical structure were considered for the analysis of the interaction with Mps1 kinase domain. In addition to genistein (CID 5280961), the molecules daidzein (CID 5281708), glycitein (CID 5317750), and fisetin (CID 5281614) were selected as the most promising flavonoids hits for molecular modeling studies of Mps1 kinase–ligand complex formation.

### 4.2. Ligand Preparation

The LigPrep module of the Maestro suite was used to select three-dimensional conformations of low-energy molecules from the aforementioned chemical database and the programme Epik was employed to predict pKa values in the 7.0–7.5 pH range. The OPLS4 force field implemented in Maestro was used for energy minimization of all compounds [71].

### 4.3. Molecular Docking Studies

#### 4.3.1. Grid Generation

Docking grids were generated using the receptor grid generation tool within Glide to ensure ligand screening was performed in the appropriate site of the Mps1 catalytic domain. This was followed by the use of the Glide module to carry out high-throughput virtual screening (HTVS) and extra precise (XP) docking.

#### 4.3.2. HTVS and XP Docking

The HTVS and XP Glide-dock modules integrated within the Schrödinger package were used for HTVS and XP docking calculations [72,73]. The ligand poses that were generated were subjected to a series of hierarchical filters to assess ligand interactions with Mps1. The OPLS4 force field was used for energy minimisation. The best scoring compounds from the HTVS were selected for further analysis with XP Glide-docking.

### 4.4. In Silico Pharmacokinetics Studies

A set of physicochemical properties were investigated on selected compounds, including pan assay interference compounds (PAINS) [55,56] calculations using QikProp integrated in Maestro; and the prediction of ADME parameters using the swissADME webserver [58].

## 5. Conclusions

In this contribution, we report the development of a computational biology approach to study the dynamics of Mps1 kinase interactions with small-sized molecules of the isoflavone class, which has led to the identification of new potential Mps1 kinase inhibitors with predicted favorable drug-like features. Further functional studies of the compounds described in this work using an experimental approach will be carried out against a panel of cancer cells of diverse tissue origin, which should pave the way to identifying lead chemotypes, and, ultimately, to developing more effective therapeutics for the treatment of cancer and possibly other malignancies associated with Mps1 overexpression, defects in chromosome segregation, cell division, and cell proliferation. In a broader sense, our study provides a perspective of how state-of-the-art computational biology approaches can be used in oncology to advance the design of more effective drugs for the treatment of cancer.

## Figures and Tables

**Table 1:** Mps1 kinase inhibitors selected for HTVS.

Compound (PubChem ID)	XP Glide Score (kcal/mol)	IC_50_ (nM)	Reference
Reversine (210332) 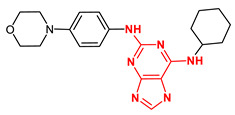	−10.97	6	[52]
Bay1217389 (78320750) 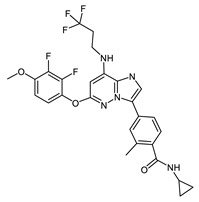	−10.18	50	[25]
Bos-172722 (73386890) 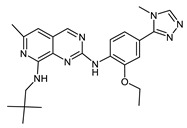	−9.05	3	[53]
Bay-1161909 (71599640) 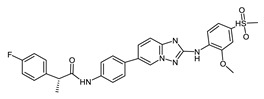	−8.17	0.34	[25]
CIF-402257 (118086034) 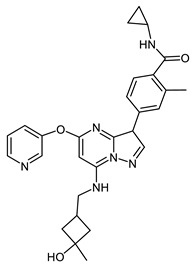	−8.0	10	[54]

**Table 2:** XP Glide docking score and type of contacts with Mps1 kinase residues of selected compounds.

Compound ID	2D Structure	Glide Score (kcal/mol)	Type of Interaction and Mps1 Residues Involved
210332 Reversine	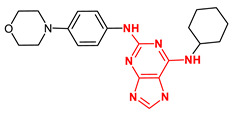	−10.97	2 H-bonds Gly605, and 1H bond Glu603
123679245	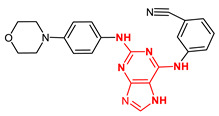	−11.10	2 H-bonds Gly605, and 1H bond Glu603
68916574	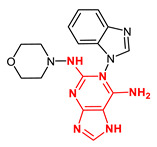	−9.34	2 H-bonds Gly605
117091061	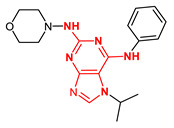	−9.23	2 H-bonds Gly605
57639384	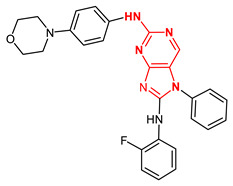	−9.2	2 H-bonds Gly605
21189870	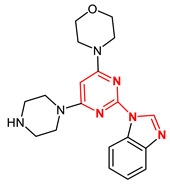	−9.0	1 H-bond Gly605

**Table 3:** Some of the key physicochemical descriptors of selected compounds that were computed with QikProp and structural alerts (PAINS).

Compound	QPlogS	QPlogHERG	QPPCaco	QPlogBB	% Human Oral Absorption	PAINS
Reversine	−5.22	−5.01	922	−0.71	100	1
123679245	−6.37	−6.29	333	−1.26	87.8	1
68916574	−4.08	−4.72	1296	−0.23	93.8	0
117091061	−4.11	−6.08	669	−0.11	95.8	0
57639384	−7.78	−7.45	2334	−0.35	100	1
21189870	−3.55	−5.57	501	+0.27	89.0	0

**Table 4:** XP GScore and physicochemical descriptors of the selected isoflavones.

COMPOUND ID	XP GScore (kcal/mol)	QPlogS	QPlogHERG	QPPCaco	QPlogBB	%(HOA)	PAINS
Fisetin	−10.27	−2.7	−4.9	52	−1.8	60	1
Genistein	−9.7	−2.9	−4.9	171	−1.2	76	0
Daidzein	−9.2	−2.9	−5.0	397	−0.9	83	0
Glycitein	−7.9	−3.2	−5.0	404	−0.9	84	0

QPlogS, predicted aqueous solubility [−6.5/0.5]; QPlogHERG, K+ channel blockage (log IC_50_) [<−5 concern]; QPPCaco, apparent Caco-2 cell permeability in nm/s [<25 poor, >500 excellent]; QPlogBB, predicted log of the brain/blood partition coefficient [−3.0/1.2]; percent of human oral absorption %(HOA) in GI [<25% is poor] [range of 95% of drugs]. PAINS corresponds to the number of structural alerts calculated with the SwissADME webserver [58].

**Table 5:** Physicochemical descriptors of the selected flavonoids by molecular docking.

Compound CID	QPlogS	QPlogHERG	QPPCaco	QPlogBB	%(HOA)	PAINS
5378180	−2.66	−4.83	14.83	−2.62	31	0
11810419	−4.09	−4.64	134.52	−1.57	79	0
24039298	−2.39	−4.74	61.39	−2.17	61.8	0

## Supplementary Materials

The following supporting information can be downloaded at: https://www.mdpi.com/article/10.3390/ijms232214228/s1.
