# Supplementary material for: Computational Biology Dynamics of Mps1 Kinase Molecular Interactions with Isoflavones Reveals a Chemical Scaffold with Potential to Develop New Therapeutics for the Treatment of Cancer
Source: Int J Mol Sci. 2022 Nov 17;23(22):14228. doi: 10.3390/ijms232214228 (PMC9692432; doi:10.3390/ijms232214228)

International Journal of
Molecular Sciences

MDPI

Support Information

The dynamics of Mps1 kinase molecular interactions with iso-flavones revealed a chemical scaffold with potential to develop new therapeutics for the treatment of cancer

Lauren Pugh[1], Alisha Pancholi[1], Priscila Celeste Purat[1], Sandra Agudo-Alvarez[2], Raúl Benito-Arenas[2], Agatha Bastida[2]*, Victor M. Bolanos-Garcia[1*]

Figure S1.- 2D structure of Reversine (A), Fisetin (B), Genistein (C), Daidzein (D) and Glycitein (E) bound to Mps1 (PDB ID 5ljj).

A

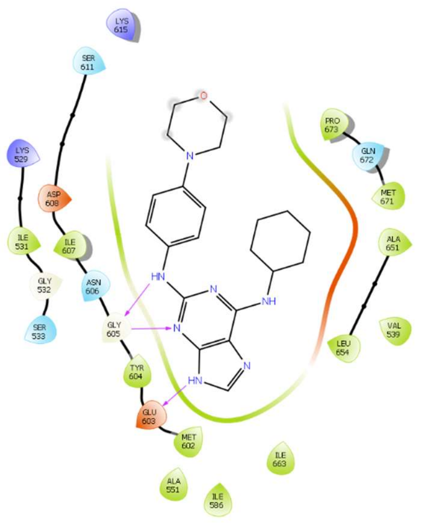

B

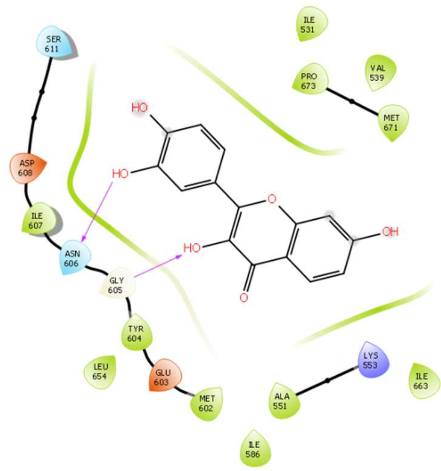

C

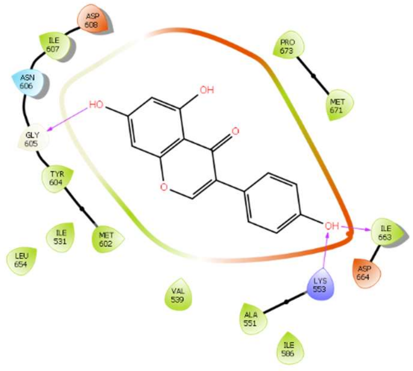

D

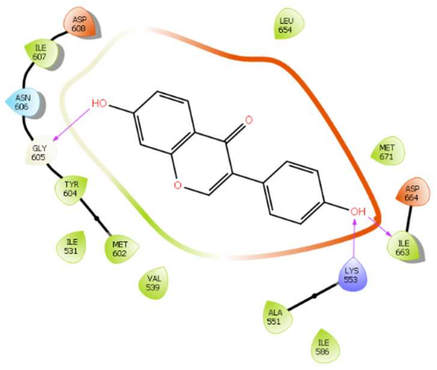

E

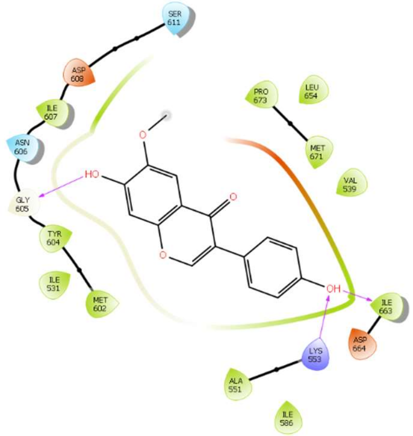

Figure S2.- (A) 2D-structure of the nucleotide binding pocket (ATP) of the Mps1; (B) 3-D representation of the Mps1-ATP complex (PDB ID 3hmn).

A B

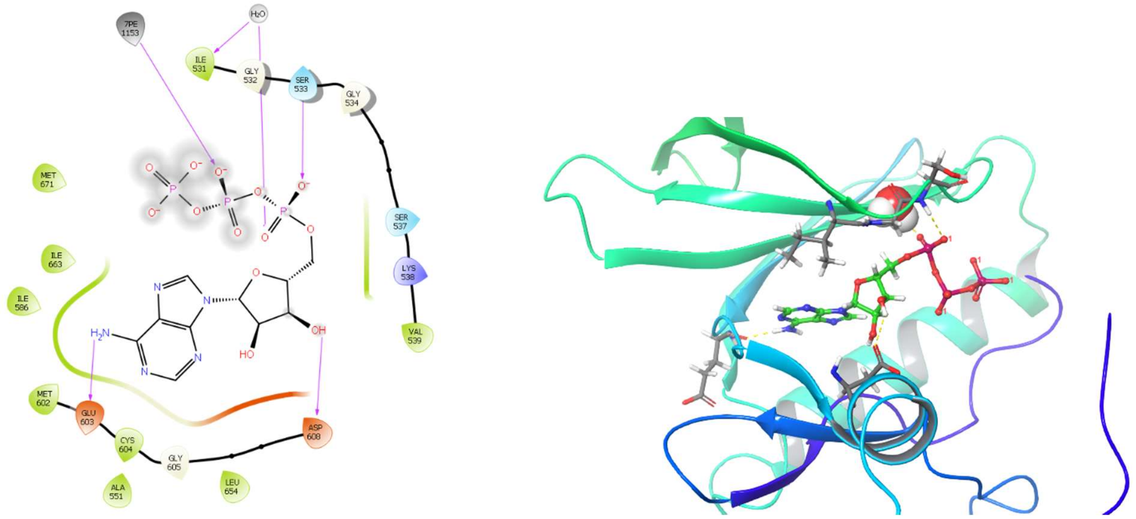

Figure S3.-2D structures of new isoflavoids bound to human Mps1 kinase domain. (A) CID 68916574; (B) CID 5378180; (C) CID 11810419; (D) CID 24039298.

A

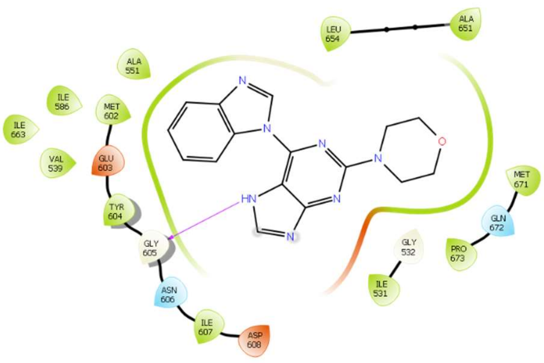

B

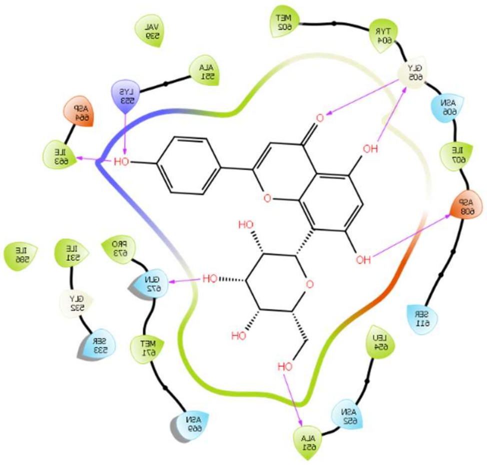

C

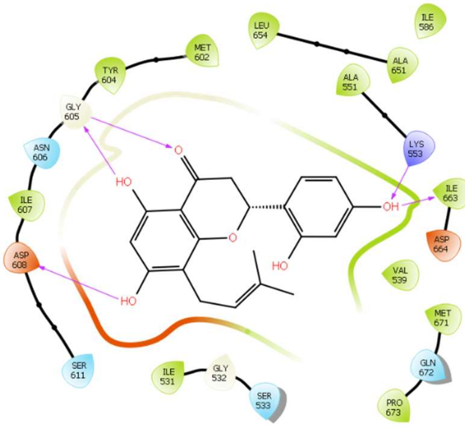

D

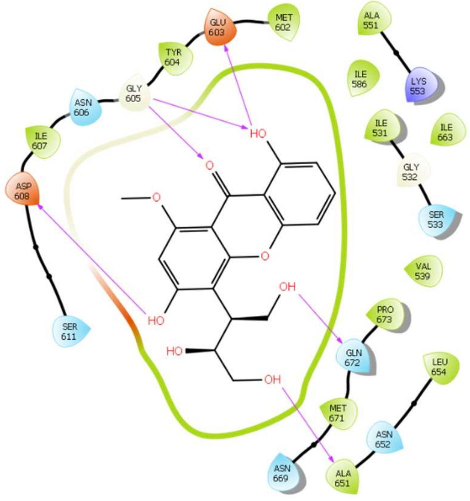

Supplement: Supplementary file 1 [file ijms-23-14228-s001.zip › ijms-2005138-supplementary.pdf]
